# Aluminum concentrations in central and peripheral areas of malignant breast lesions do not differ from those in normal breast tissues

**DOI:** 10.1186/1471-2407-13-104

**Published:** 2013-03-08

**Authors:** Raquel Mary Rodrigues-Peres, Solange Cadore, Stefanny Febraio, Juliana Karina Heinrich, Katia Piton Serra, Sophie F M Derchain, Jose Vassallo, Luis Otavio Sarian

**Affiliations:** 1 Department of Obstetrics and Gynecology, Faculty of Medical Sciences-University of Campinas, Campinas, Brazil; 2 Department of Analytical Chemistry, Institute of Chemistry-University of Campinas, Campinas, SP, Brazil; 3 Department of Pathology, Faculty of Medical Sciences-University of Campinas, Campinas, Brazil

**Keywords:** Aluminum, Breast, Cancer, Atomic spectrometry, Biohazard

## Abstract

**Background:**

Aluminum is used in a wide range of applications and is a potential environmental hazard. The known genotoxic effects of aluminum might play a role in the development of breast cancer. However, the data currently available on the subject are not sufficient to establish a causal relationship between aluminum exposure and the augmented risk of developing breast cancer. To achieve maximum sensitivity and specificity in the determination of aluminum levels, we have developed a detection protocol using graphite furnace atomic absorption spectrometry (GFAAS). The objective of the present study was to compare the aluminum levels in the central and peripheral areas of breast carcinomas with those in the adjacent normal breast tissues, and to identify patient and/or tumor characteristics associated with these aluminum levels.

**Methods:**

A total of 176 patients with breast cancer were included in the study. Samples from the central and peripheral areas of their tumors were obtained, as well as from the surrounding normal breast tissue. Aluminum quantification was performed using GFAAS.

**Results:**

The average (mean ± SD) aluminum concentrations were as follows: central area, 1.88 ± 3.60 mg/kg; peripheral area, 2.10 ± 5.67 mg/kg; and normal area, 1.68 ± 11.1 mg/kg. Overall and two-by-two comparisons of the aluminum concentrations in these areas indicated no significant differences. We detected a positive relationship between aluminum levels in the peripheral areas of the tumors, age and menopausal status of the patients (*P* = .02).

**Conclusions:**

Using a sensitive quantification technique we detected similar aluminum concentrations in the central and peripheral regions of breast tumors, and in normal tissues. In addition, we did not detect significant differences in aluminum concentrations as related to the location of the breast tumor within the breast, or to other relevant tumor features such as stage, size and steroid receptor status. The next logical step is the assessment of whether the aluminum concentration is related to the key genomic abnormalities associated with breast carcinogenesis.

## Background

The use of aluminum, in many different chemical presentations, has now reached the highest level in documented history. This element is one of the most common metals in the lithosphere, and it is utilized in a wide range of industries manufacturing products such as food, paper, dyes, pigments, paints, glass, fuels, textiles, cosmetics and pharmaceuticals; it is also used in water purification and oil refining processes. The presence of elevated concentrations of aluminum in the general public might be a consequence of its extensive use [1].

It has been hypothesized that powerful antiperspirants containing aluminum compounds, widely used in current formulations of hygiene products that are generally applied to the axilla, may pose some risks to health [2]. Recent evidence has indicated increased genomic instability in the outer quadrants of the breast [3], and in one report it was suggested that higher levels of aluminum may be present in this region (axilla and lateral) relative to the inner breast regions (medial and middle) [4]. Besides being associated with carcinogenic effects in animal studies, aluminum is known to bind to DNA and to be genotoxic. The metal also exhibits neuronal effects in humans, showing an influence on iron homeostasis, which might link aluminum chronic exposure with the development of Parkinson’s and Alzheimer’s diseases [5,6]. Also, a few types of metal such as aluminum, cadmium, mercury, copper, cobalt, among others, have the capacity to bind to the estrogen receptors in cells and mimic the function of this hormone, although other studies have refuted this idea [7-9]. Even though they have different and complex structures, some of these metals were described as having the ability to bind to estrogen receptors in the majority of tumor cell lines tested, both *in vitro* and *in vivo*; this mechanism might lead to altered protein expression, mammary gland development and precocious puberty, and can increase the height of the uterus and influence androgen response [7].

The current data regarding elevated levels of aluminum in some areas of the breast, and the known biological effects of this metal in breast tissues, are not sufficient to establish a causal relationship between aluminum exposure and the augmented risk of developing breast cancer. Studies addressing this issue differ in relation to the techniques used to detect and quantify the aluminum levels.

High specificity techniques, formerly used in non-biological experiments, have now been standardized for metal level determinations in biological samples [10-12]. Atomic Absorption Spectrometry (AAS) has been used to accurately determine the metal concentrations in human breast tissues [4]. In one study using this technique, significantly higher levels of a few heavy metals were detected in the blood and tissues of women with breast lesions relative to healthy controls [13].

We have developed an aluminum detection protocol, tailored for breast tissues, using graphite furnace-AAS (GFAAS) to achieve maximum sensitivity and specificity in the determination of aluminum levels. In the present study, we thus contrasted the aluminum levels of central and peripheral areas of breast carcinomas and the adjacent normal breast tissues in an unprecedentedly large set of patients. We also tried to identify patient and/or tumor characteristics that were possibly associated with aluminum levels.

## Methods

### Patients and sample collection

**Figure 1:**
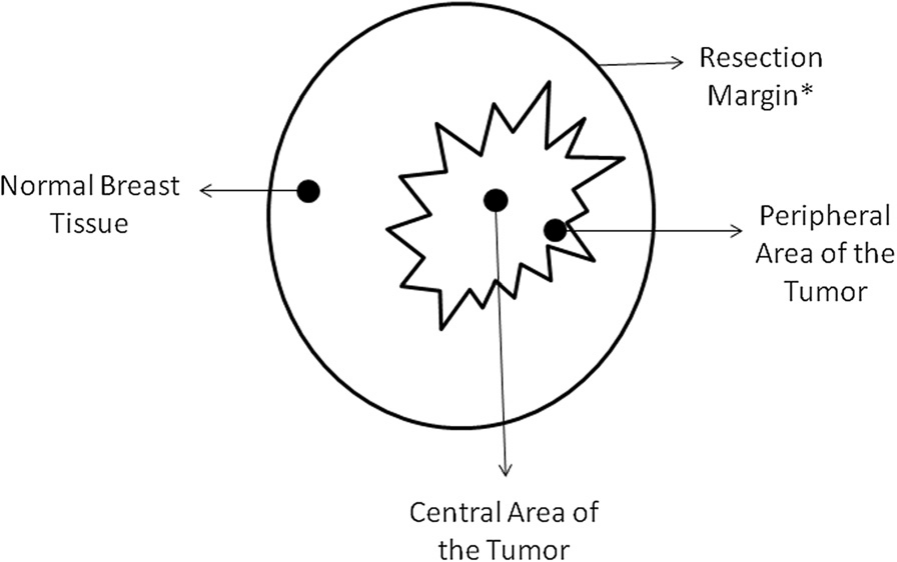
**Diagram showing the locations of the tissue samples obtained from each subject.** Surgical specimens and lesion dimensions vary from subject to subject. *Resection margins apply to conservative surgeries (quadrantectomies). For patients treated with radical mastectomy, the resection margins are the boundaries of the resected organ.

For this cross-sectional study, we recruited a total of 176 women who had consecutively undergone surgical (either radical or conservative) treatment for breast cancer at the Breast Cancer Clinics of the Women’s Hospital Prof. Dr. José Aristodemo Pinotti - CAISM, at the State University of Campinas (UNICAMP), between 2008 and 2010. Immediately after the removal of the surgical specimen one of the researchers macroscopically assessed the specimen, identified and measured the tumor area. Next, if the tumor area had a great axis that was larger than 1.0 cm, the researcher sampled the central and the peripheral regions of the tumor with a scalp, and divided each of the two samples into two mirror fragments. Also, a sample from a macroscopically normal glandular area of the breast was obtained, and the sample was divided into two mirrored fragments (Figure 1). One of the two fragments from the central and peripheral tumor areas, as well as from the normal glandular area, were stored at −196°C for further measurements of aluminum concentration, and the other fragment was fixed in formalin for subsequent embedding in paraffin. The fragment sent for paraffin embedment was processed and stained using H&E, and subsequently assessed by an experienced pathologist who determined the presence of invasive carcinomas in the sample. The mirror fragments of the selected specimens were sent for aluminum quantification. Twenty-six women had to be excluded from this study due to technical difficulties or the absence of invasive breast carcinoma in the collected fragment. Thus, 150 viable samples were obtained for the study. Clinicopathological data were obtained from patient records. The study protocol was fully approved by the Research Ethics Committee of the Faculty of Medical Sciences - State University of Campinas (CEP #705/2007) and all patients signed informed consent forms.

### Aluminum quantification using GFAAS

Evaluation of tissue aluminum content was carried out at the Chemistry Institute of the Campinas State University (IQ/UNICAMP). The previously frozen tissue samples (−196°C) from the central and peripheral areas of the tumors, and also from adjacent areas of normal tissue, were dried in Falcon tubes in a vacuum desiccator at low pressure for 60 h. These tubes were weighed before and after tissue drying, for estimation of the wet mass. After drying, the tubes containing the samples were weighed to determine the dry tissue mass. This enabled standardization of the amount of tissue used in each case, thus removing the interference from water content. All of the glassware used in the analysis was previously treated with 10% v/v concentrated HNO_3_ and then washed three times with ultrapure water. Sample solubilization was carried out overnight using 25% (m/v) tetramethylammonium hydroxide (TMAH), in the proportion of 1 ml of reagent to 250 mg of wet sample [14]. After solubilization, the samples were diluted with an aqueous solution of 0.35% Triton-X 100 to a final volume corresponding to a dilution factor of 75 times; this was prepared with ultrapure water for stabilization of the fat/water system, and the samples were analyzed using GFAAS.

The measurements were carried out using the following: a GFAAS (AAnalyst model 600, Perkin-Elmer, Norwalk, CT, USA) with background correction based on the Zeeman effect; an automatic sampler (model AS-800, Perkin-Elmer, Norwalk, CT, USA); and THGA graphite tubes with an integrated L’vov platform and transversal heating (Perkin-Elmer). An aluminum hollow cathode lamp (λ = 309.271 nm; I = 25 mA) was used, and the measurements were made in integrated absorbance units. The volumes of the sample and the chemical modifier, Mg(NO_3_)_2_, were 20 μL and 5 μL, respectively. External calibration standards of aluminum containing 1.3% TMAH solution (v/v), which comprised aluminum concentrations from 2–24 μg/kg, were used under the optimized instrumental conditions for the heating program shown in Table 1. The samples analyzed were fat-rich and this is undesirable in order to have quantitative recovery of aluminum during GFAAS measurements. In this case, an alkaline treatment with TMAH [14] allowed the complete solubilization of the samples. Additionally, the use of (Mg(NO_3_)_2_) as chemical modifier for the GFAAS heating program showed to be mandatory to obtain quantitative recoveries of the analyte. The correlation coefficients (R) for aluminum concentration and the water content of the samples were: 0.23 for normal tissues; 0.26 for peripheral tumor areas, and 0.16 for peripheral tumor areas, showing that samples with increased water content (and therefore lower fat content) had slightly higher concentrations of aluminum (Table 1). Analytical curves with correlation coefficient values lower than 0.995 were not accepted. Samples were divided into five smaller groups for analysis; for each group two reagent blanks were prepared, ensuring that the contamination originating from reagents and from the laboratory environment was minimized. Aluminum measurements were displayed in mg/kg. All of the measurements were made in triplicate. Analytical curve plots and calculation of aluminum concentration were carried out using ORIGIN PRO 7.0 software. To verify the accuracy of the proposed method, experiments involving the addition and recovery of the analyte were conducted, showing values between 96 and 111%.

**Table 1:** Optimized heating program for GF AAS measurements

***Step***	***Temperature***	***Ramp Time***	***Hold time***	***Flow rate***
	***(°C)***	***(s)***	***(s)***	***(mL min***^***− 1***^***)***
Drying	110	1	30	250
Drying	130	15	30	250
Pyrolysis	1500	10	20	250
Atomization	2350	0	5	0
Cleaning	2450	1	3	250
Cooling	20	1	5	250

Note: Correlation coefficients for Aluminum concentration and the water content of the samples were: 0.23 for normal tissues; 0.26 for peripheral tumor areas, and 0.16 for peripheral tumor areas, showing that samples with increased water content (and therefore lower fat content) had slightly higher concentrations of aluminum.

### Statistical analysis

Data were stored in Excel® spreadsheets and analyzed using the R Environment for Statistical Computing [15]. Confidence levels were set at 95% (*P* < .05 was considered significant). As a first step, we tested whether aluminum concentrations in the three regions (central and peripheral tumor regions, and normal breast tissues) conformed to the assumption of normality using the Shapiro-Wilk test. Log-transformed (to base e) values were used, since the raw data showed marked skewness. Results are presented in the original scale. After ascertaining data compliance to normality, we used the pairwise t-test for the comparison of the aluminum concentration in the central, peripheral and normal areas of the tumors. Paired comparisons were used in the calculations regarding data from central, peripheral and normal tissue samples obtained from the same individual. Next, we assessed the relationship between the clinical and pathological features of the cases and the aluminum concentration in the central, peripheral and normal areas (Tables 2, 3 and 4). For these analyses, we first examined the distribution of the aluminum concentration in the whole set of samples, considering the tissue’s dry and defatted mass. We compared the mean aluminum concentration in relation to the clinical and pathological features of the patients and tumors (age, body mass index, menopausal status, quadrant harboring the tumor, tumor size, disease stage, histological grade, estrogen/progesterone receptor and cerbb2 statuses) using a linear regression model, for which we used log-transformed (to base e) data. We next determined three aluminum concentration groups based on the approximate three-tiered percentile distribution of the data: negative (null aluminum readings); 0.05-2.0 mg/kg; and ≥ 2.0 mg/kg. Finally, we tested whether the clinical and pathological features of the patients and tumors features were associated with the trend in aluminum concentration using the chi-squared proportion trend test.

**Table 2:** Aluminum content in central areas of the tumor according to the clinical characteristics of the women and the pathological features of the tumors

***Characteristics***	***Total***	***Aluminum content in mg/Kg***								
		**Mean (sd)**		Negative*		**0.05 - 2.0**		**≥ 2**		
***Age***			P**	n	(%)	n	(%)	n	(%)	P trend***
**<40**	20	1.25(1.49)	0.12	6	(17.6)	9	(11.1)	5	(14.3)	0.68
**>40**	130	1.97(3.81)		28	(82.4)	72	(88.9)	30	(85.7)	
***BMI***										
**<25**	46	1.96(2.43)	0.74	7	(28)	26	(38.2)	13	(40.6)	0.34
**≥25**	79	2.05(4.39)		18	(72)	42	(61.8)	19	(59.4)	
***Menopausal status***										
**Post**	42	1.28(1.32)	**0.03**	9	(33.3)	23	(32.9)	10	(29.4)	0.73
**Pre**	89	2.34(4.40)		18	(66.7)	47	(67.1)	24	(70.6)	
***Quadrant***										
**Upper external**	92	1.83(3.61)	0.93	23	(67.6)	47	(58)	22	(62.9)	0.69
**Other**	58	1.94(3.61)		11	(32.4)	34	(42)	13	(37.1)	
***Tumor size***										
**<2.0 cm**	72	2.36(4.64)	0.21	13	(38.2)	39	(49.4)	20	(57.1)	0.11
**≥2.0 cm**	76	1.44(2.21)		21	(61.8)	40	(50.6)	15	(42.9)	
***Stage***										
**I-II**	90	2.19(4.37)	0.13	19	(57.6)	49	(61.3)	22	(62.9)	0.65
**III – IV**	58	1.42(1.90)		14	(42.4)	31	(38.8)	13	(37.1)	
***Histologic grade***										
**I-II**	26	2.36(6.02)	0.79	5	(15.6)	17	(23)	4	(12.1)	0.70
**III**	113	1.74(2.87)		27	(84.4)	57	(77)	29	(87.9)	
***Estrogen receptor***										
**Negative**	40	1.34(1.48)	0.67	12	(36.4)	17	(22.4)	11	(32.4)	0.72
**Positive**	103	2.13(4.22)		21	(63.6)	59	(77.6)	23	(67.6)	
***Progesterone receptor***										
**Negative**	56	1.34(1.31)	0.86	15	(45.5)	28	(36.8)	13	(39.4)	0.61
**Positive**	86	2.25(4.59)		18	(54.5)	48	(63.2)	20	(60.6)	
***cerbb2 status***										
**Negative**	94	1.91(3.54)	0.18	22	(66.7)	47	(63.5)	25	(75.8)	0.43
**Positive**	46	1.91(4.07)		11	(33.3)	27	(36.5)	8	(24.2)	

**Note:** The aluminum concentration was categorized as negative, 0.05-2.0 mg/ kg and ≥ 2.0 mg/ kg according to the three-tiered percentile distribution of the raw data. *Aluminum detection threshold for the GF-AAS was set at 0.05 mg/kg; ** In this column, the p values refer to the comparison of the mean Aluminum concentrations in each strata, using independent samples t-tests with the log-transformed (to base e) data; ***In this column, the p-values refer to the Chi-squares for trends; i.e. if p ≤ 0.05, there is a significant trend in Aluminum concentration related to the specified characteristic (see statistics for more details).

**Table 3:** Aluminum content in peripheral areas of the tumor according to the clinical characteristics of the women and the pathological features of the tumors

***Characteristics***	***Total***	***Aluminum content in mg/Kg***								
		***Mean (sd)***		Negative*		**0.05 - 2.0**		**≥ 2**		
***Age***			P**	n	(%)	n	(%)	n	(%)	P trend***
**<40**	20	1.71(1.79)	**0.02**	4	(16)	9	(10)	7	(20)	0.52
**>40**	130	1.97(3.81)		21	(84)	81	(90)	28	(80)	
***BMI***										
**<25**	46	2.13(1.06)	0.87	5	(31.2)	28	(36.8)	13	(39.4)	0.59
**≥25**	79	2.34(1.93)		11	(68.8)	48	(63.2)	20	(60.6)	
***Menopausal status***										
**Post**	42	2.09(2.33)	**0.02**	6	(35.3)	22	(27.8)	14	(40)	0.49
**Pre**	89	2.47(1.15)		11	(64.7)	57	(72.2)	21	(60)	
***Quadrant***										
**Upper external**	92	1.96(3.57)	0.86	14	(56)	56	(62.2)	22	(62.9)	0.61
**Other**	58	2.33(1.97)		11	(44)	34	(37.8)	13	(37.1)	
***Tumor size***										
**<2.0 cm**	72	2.31(7.23)	0.81	12	(48)	40	(44.9)	20	(58.8)	0.34
**≥2.0 cm**	76	1.92(3.77)		13	(52)	49	(55.1)	14	(41.2)	
***Stage***										
**I-II**	90	1.76(3.23)	0.48	13	(52)	56	(63.6)	21	(60)	0.61
**III – IV**	58	2.67(2.19)		12	(48)	32	(36.4)	14	(40)	
***Histologic grade***										
**I-II**	26	1.28(1.17)	0.83	5	(21.7)	16	(19.3)	5	(15.2)	0.52
**III**	113	2.40(2.48)		18	(78.3)	67	(80.7)	28	(84.8)	
***Estrogen receptor***										
**Negative**	40	1.77(1.57)	0.19	5	(21.7)	22	(25.6)	13	(38.2)	0.14
**Positive**	103	2.32(2.76)		18	(78.3)	64	(74.4)	21	(61.8)	
***Progesterone receptor***										
**Negative**	56	1.94(2.56)	0.15	7	(30.4)	32	(37.6)	17	(50)	0.12
**Positive**	86	2.31(7.20)		16	(69.6)	53	(62.4)	17	(50)	
***cerbb2 status***										
**Negative**	94	1.91(3.54)	0.09	17	(73.9)	52	(62.7)	25	(73.5)	0.86
**Positive**	46	1.91(4.07)		6	(26.1)	31	(37.3)	9	(26.5)	

**Note:** The aluminum concentration was categorized as negative, 0.05-2.0 mg/ kg and ≥ 2.0 mg/ kg according to the three-tiered percentile distribution of the raw data. *Aluminum detection threshold for the GF-AAS was set at 0.05 mg/kg; ** In this column, the p values refer to the comparison of the mean Aluminum concentrations in each strata, using independent samples t-tests with the log-transformed (to base e) data; ***In this column, the p-values refer to the Chi-squares for trends; i.e. if p ≤ 0.05, there is a significant trend in Aluminum concentration related to the specified characteristic (see statistics for more details).

**Table 4:** Aluminum content in normal areas of the breast according to the clinical characteristics of the women and the pathological features of the surrounding tumors

***Characteristics***	***Total***	***Aluminum content in mg/Kg***								
		**Mean (sd)**		Negative*		**0.05 - 2.0**		**≥ 2**		
***Age***			P**	n	(%)	n	(%)	n	(%)	P trend***
**<40**	20	1.13(1.59)	0.17	3	(14.3)	15	(12.8)	2	(16.7)	0.91
**>40**	130	1.76(11.90)		18	(85.7)	102	(87.2)	10	(83.3)	
***BMI***										
**<25**	46	3.68(19.9)	0.38	5	(33.3)	36	(35.6)	5	(55.6)	0.40
**≥25**	79	0.78(1.13)		10	(66.7)	65	(64.4)	4	(44.4)	
***Menopausal status***										
**Post**	42	4.06(20.88)	0.12	6	(35.3)	33	(31.7)	3	(30)	0.75
**Pre**	89	0.73(0.98)		11	(64.7)	71	(68.3)	7	(70)	
***Quadrant***										
**Upper external**	92	2.19(14.13)	0.59	15	(71.4)	70	(59.8)	7	(58.3)	0.37
**Other**	58	0.87(1.32)		6	(28.6)	47	(40.2)	5	(41.7)	
***Tumor size***										
**<2.0 cm**	72	0.78(0.94)	0.28	6	(30)	60	(51.7)	6	(50)	0.16
**≥2.0 cm**	76	2.56(15.56)		14	(70)	56	(48.3)	6	(50)	
***Stage***										
**I-II**	90	2.19(14.28)	0.42	10	(50)	74	(63.8)	6	(50)	0.75
**III – IV**	58	0.92(1.51)		10	(50)	42	(36.2)	6	(50)	
***Histologic grade***										
**I-II**	26	0.89(1.30)	0.53	4	(21.1)	20	(18.2)	2	(20)	0.88
**III**	113	1.89(12.75)		15	(78.9)	90	(81.8)	8	(80)	
***Estrogen receptor***										
**Negative**	40	0.56(0.49)	**0.009**	2	10	37	33.3	1	(8.3)	0.62
**Positive**	103	2.18(13.37)		18	90	74	66.7	11	(91.7)	
***Progesterone receptor***										
**Negative**	56	0.63(0.68)	**0.04**	4	(20)	49	(44.5	3	(25)	0.43
**Positive**	86	2.46(14.62)		16	(80)	61	(55.5)	9	(75)	
***cerbb2 status***										
**Negative**	94	0.82(1.19)	0.77	13	(68.4)	74	(67.9)	7	(58.3)	0.61
**Positive**	46	3.68(19.96)		6	(31.6)	35	(32.1)	5	(41.7)	

Note: The aluminum concentration was categorized as negative, 0.05-2.0 mg/ kg and ≥ 2.0 mg/ kg according to the three-tiered percentile distribution of the raw data. *Aluminum detection threshold for the GF-AAS was set at 0.05 mg/kg; ** In this column, the p values refer to the comparison of the mean Aluminum concentrations in each strata, using independent samples t-tests with the log-transformed (to base e) data; ***In this column, the p-values refer to the Chi-squares for trends; i.e. if p ≤ 0.05, there is a significant trend in Aluminum concentration related to the specified characteristic (see statistics for more details).

## Results

Table 5 shows the aluminum concentration per tumor region, taking into consideration the central and peripheral regions of the tumor and normal breast tissue. The average aluminum content was 1.88 mg/kg in the central areas, 2.10 mg/ kg in the peripheral areas and 1.68 mg/ kg in the normal areas. Overall and two-by-two comparisons of the aluminum concentrations in these areas disclosed no significant differences. The average aluminum content in tumor areas (either central or peripheral) was not significantly different from that in normal tissues.

**Table 5:** Comparison of Aluminum concentration in central and peripheral regions of the tumors, and normal breast tissue

**Aluminum concentration per tumor region (mg/kg)**				
	**Central**	**Peripheral**	**Normal**	
Mean	1.88	2.10	1.68	p-value*
Standard deviation	3.60	5.67	11.1	0.88
	**Cancer regions grouped**		**Normal**	
Mean	1.99		1.68	p-value*
Standard deviation	3.46		11.1	0.74
**Two-by-two comparisons of the tumor regions**	**Mean difference**		p-value*	
Central-Peripheral	0.67		0.96	
Central-Normal	0.83		0.97	
Peripheral-Normal	0.67		0.87	

*Paired t-tests were used to evaluate the within-individual variations in Aluminum concentrations. Log-transformed (to base e) data were used (see statistics section) in order to conform to normality.

The average aluminum concentration in each area was analyzed according to the key clinical features and pathological characteristics of the cases (Tables 2, 3 and 4). We detected a positive relationship between aluminum levels in the peripheral areas of the tumors, age and menopausal status of the patients (*P* = .02 in each case) and in the central areas for menopausal status (p=.03). We also detected a positive relationship between estrogen and progesterone receptor expression and aluminum levels in the normal tissues (p = 0.009 and 0.04, respectively). The other comparisons did not yield any significant results. Next, the aluminum content was categorized into three levels (negative, 0.05-2.0 mg/kg and > 2.0 mg/kg), respecting the three-tiered percentile distribution of aluminum concentration in the whole. No significant trends in patient or tumor characteristics were found as related to the aluminum concentrations.

## Discussion

In our study, using a high-specificity technique, we detected similar aluminum concentrations in the central and peripheral regions of breast tumors. More interestingly, these concentrations did not depart from the aluminum levels found in the surrounding normal tissues. The results clearly suggest that there is no aluminum gradient from normal to diseased breast tissue. Our study also examined whether tumor location within the breast, among other variables, influenced the aluminum concentrations; results were negative.

The relationship between aluminum exposure and breast cancer has been hypothesized for a long time. However, there are technical challenges involved in measuring the metal content of human tissues. This is especially true for breast tissues which contain a high content of fat, which makes sample preparation difficult. Early studies used indirect measurements of the aluminum content of breast tissues, for example questionnaires addressing the use of antiperspirants containing aluminum [16,17]. More recent studies have used better performing direct measurement techniques [18,19]. Even minimal differences in aluminum concentration may be of biological significance, since *in vitro* studies regarding the effects of aluminum and other metals on the cell have demonstrated that even concentrations as low as 4 nmol/g may exert proestrogenic and possibly carcinogenic effects [4]. Our study was a specificity-driven attempt at quantifying the aluminum content of the human breast, and featured one of the larger sample sizes of diseased and normal breast tissues. Pasha et al. [19] compared malignant and benign breast lesions (normal breast tissue was not examined) in a set of 114 lesions, and found no difference in aluminum concentration between benign and malignant lesions. In another study [4], the aluminum content was measured in tissue samples from the axilla, lateral, middle and medial regions of the breast of 17 patients who underwent mastectomy due to breast cancer. These authors reported a significantly higher aluminum concentration in the outer (axilla plus lateral regions) relative to the inner (middle and medial regions) breast. By way of contrast, the aluminum concentration did not differ across breast quadrants in our study. However, it is worth mentioning that it was designed to evaluate differences in aluminum concentrations between central and peripheral areas of breast tumors, but not to assess the differences in aluminum concentrations across breast quadrants.

As compared with other studies, we detected lower aluminum concentrations in breast cancer tissues and normal breast samples. Differences related to sample collection and processing might have accounted for the different results. While a majority of studies have reported mean values of aluminum for both normal and cancerous tissues in the range 3.63-19.5 μg/g [18-20], our results showed values of 1.68 μg/g for normal breast specimens and 1.88-2.1 μg/g for breast cancer specimens (when converted to the same unit of measurement).

We detected a non-significant trend in aluminum concentration from the inner core of the tumors to the peripheral areas. Speculatively, this trend may be attributable to the preferential accumulation of aluminum due to the biochemistry of tumorous tissues, as previously hypothesized by Mannello [21]. It is also sensible to infer that the outer regions of the tumor may concentrate higher quantities of aluminum due to uptake by the immune system from the axillary skin.

We found a significant association between age and menopausal status with aluminum concentration in the central and peripheral regions of the tumors. This may be attributable to the time effect of continual usage of aluminum-based antiperspirants, or to other exposures. However, this finding has never been reported before for breast tissues, although other studies using nipple aspirate fluids from women with breast cancer have shown higher aluminum levels in post-menopausal subjects. Also, it has been suggested that age might be related to aluminum accumulation over time [2,21,22].

Our study had a few possible technical shortcomings. Of particular concern is the possible interaction between aluminum and other trace elements, such as calcium. It is known that the calcium binding protein osteopontin forms stable complexes with aluminum. It has also been established that both malignant and nonmalignant breast lesions frequently present with microcalcifications, which may in turn also harbor significant amounts of calcium [21,23,24]. We did not control our aluminum measurements for calcium concentrations in the sample, or recorded the number of radiologically detectable microcalcifications in the breast lesions. It has also been demonstrated that aluminum influences the homeostasis of iron binding proteins, in the form of ferritin and transferrin [21].

## Conclusion

In conclusion, our study clearly suggests that the aluminum content of breast tumors may not differ significantly from that of the surrounding normal areas of the breast. In addition, we did not detect significant differences in aluminum concentrations as related to the location of the breast tumor within the breast, or to other relevant tumor features such as stage and size. The next logical step is the assessment of whether or not the aluminum concentration is related to the key genomic abnormalities associated with breast carcinogenesis, which is currently ongoing in our research laboratories.

## Competing interests

The authors declare that they have no competing interests.

## Authors’ contributions

RMRP and SF conducted the experiments and prepared the manuscript, under the supervision of LOS and SC. JH gave technical advice and KPS provided clinical advice during manuscript preparation. JV contributed on pathological advice. SD revised the final text and provided clinical advice. All authors read and approved the final manuscript.

## Pre-publication history

The pre-publication history for this paper can be accessed here:

http://www.biomedcentral.com/1471-2407/13/104/prepub
